# Mechanisms linking brain insulin resistance to Alzheimer's disease

**DOI:** 10.1590/1980-57642015DN92000003

**Published:** 2015

**Authors:** Maria Niures P.S. Matioli, Ricardo Nitrini

**Affiliations:** 1 Pós-graduanda, nível de Doutorado, Departamento de Neurologia da Faculdade de Medicina da Universidade de São Paulo.; 2 Professor Titular da Disciplina de Neurologia da Faculdade de Medicina da Universidade de São Paulo. Orientador e Professor Responsável pela Pós-graduação do Departamento de Neurologia da Faculdade de Medicina da Universidade de São Paulo.

**Keywords:** Alzheimer's disease, diabetes mellitus, insulin resistance, neurodegeneration, mechanisms

## Abstract

Several studies have indicated that Diabetes Mellitus (DM) can increase the risk of developing Alzheimer's disease (AD). This review briefly describes current concepts in mechanisms linking DM and insulin resistance/deficiency to AD. Insulin/insulin-like growth factor (IGF) resistance can contribute to neurodegeneration by several mechanisms which involve: energy and metabolism deficits, impairment of Glucose transporter-4 function, oxidative and endoplasmic reticulum stress, mitochondrial dysfunction, accumulation of AGEs, ROS and RNS with increased production of neuro-inflammation and activation of pro-apoptosis cascade. Impairment in insulin receptor function and increased expression and activation of insulin-degrading enzyme (IDE) have also been described. These processes compromise neuronal and glial function, with a reduction in neurotransmitter homeostasis. Insulin/IGF resistance causes the accumulation of AβPP-Aβ oligomeric fibrils or insoluble larger aggregated fibrils in the form of plaques that are neurotoxic. Additionally, there is production and accumulation of hyper-phosphorylated insoluble fibrillar tau which can exacerbate cytoskeletal collapse and synaptic disconnection.

## Abstract (pt)

Atualmente, muitos estudos têm indicado que o Diabetes Mellitus (DM) pode aumentar o risco de desenvolver doença de Alzheimer (DA). Esta revisão tem o objetivo de descrever brevemente os conceitos atuais sobre os mecanismos que associam DM, resistência/deficiência de insulina à DA. Resistência à insulina/fator de crescimento similar à insulina (IGF) pode contribuir para a neurodegeneração através de vários mecanismos os quais envolvem: déficit metabólico e energético, prejuízo na função do transportador de glicose-4, estresse oxidativo e do retículo endoplasmático, disfunção mitocondrial, acúmulo de AGEs, ROS e RNS com aumento na produção da neuro-inflamação e ativação da cascata pró-apoptóptica. Prejuízo na função do receptor de insulina, aumento na expressão e ativação da enzima de degradação da insulina (EDI) também têm sido descritos. Esses processos comprometem a função neuronal e glial, com redução da homeostase de neurotransmissor. Resistência à insulina/IGF causa acúmulo de fibrilas de oligômeros de PPβA-βA e grandes agregados fibrilares insolúveis em forma de placas que são neurotóxicos. Adicionalmente, há produção e acúmulo de fibrilas insolúveis de tau hiperfosforilada que podem exacerbar o colapso do citoesqueleto e a desconexão sináptica.

## INTRODUCTION

Population aging is a global phenomenon leading to an increase in chronic diseases such as dementia and diabetes mellitus (DM), which pose an epidemic challenge to global health care systems. In 2012, the WHO published that 35.6 million people had dementia worldwide and that this number is set to reach 65.7 million by 2030.^1^ Alzheimer's disease (AD) is the most common cause of dementia, especially in the elderly population.^1^ Recently, the International Diabetes Federation^2^ estimated that 382 million people had diabetes in 2013, where this number may rise to 592 million within less than 25 years.^2^ Moreover, 80% of the total number affected live in low- and middle-income countries and Type 2 diabetes (T2DM) is the most common type of DM.^2^ The prevalence of AD and T2DM increases with aging.^3^

The AD pathology is characterized by the accumulation of the following in the brain: amyloid beta precursor protein (AβPP)-Aβ large insoluble fibrillar aggregates in the form of plaques, soluble neurotoxic oligomeric fibrils, hyper-phosphorylation of tau protein with neurofibrillary tangles (NFTs) deposition, dystrophic neuritis, and neuropil threads.^4,5^ In familial forms of AD, the mutations in AβPP, presenilin 1 (PS1) and 2 (PS2) genes, or inheritance of the Apolipoprotein E e^4^ (ApoE-e4) allele can cause increased synthesis and deposition of AβPP- Aβ.^6,7^ However, the cause of AβPP-Aβ accumulation in sporadic AD, the most common form of the disease, remains unknown.^5^ However, evidence suggests that impairment in insulin and insulin-like growth factor (IGF) compromises AβPP expression and protein processing which could be responsible for AβPP-Aβ accumulation.^8^

The association between DM and AD is controversial in literature.^9,10^ Many studies have demonstrated a positive association between DM and AD, especially in epidemiological research, studies in animals and cells,^11-17^ but these findings have not been entirely confirmed in neuropathological studies.^3,18-23^ Based on this positive association, researchers have studied DM treatments as a target to diminish or avoid AD onset and progression.^24-28^

The exact mechanisms by which DM affects the brain remain unclear, but this probably occurs through cerebrovascular and neurodegenerative changes.^29^ The aim of this article was to provide a brief review on the main mechanisms associating AD with DM due to insulin resistance and deficiency.

**Insulin and insulin-like growth factor actions in the central nervous system.** The insulin produced by the pancreas can cross the blood brain barrier (BBB) from the circulation to the brain by a receptor-dependent mechanism,^30^ but the levels of insulin expression in the brain are modest compared to circulating levels.^10^ The transport of peripheral insulin across the BBB and the consequences of peripheral hyperinsulinemia or hypoinsulinemia are significantly important to cerebral insulin signaling.^10^ Insulin binding activity has been identified in the brain in a number of species, including humans.^31,32^ Furthermore, insulin receptors (IR) are expressed in cerebral vasculature and can mediate insulin traffic across the BBB.^33^

Insulin and IGF play an important role in brain function and structure.^5^ Insulin, IGF-1 and IGF-2 polypeptides and receptor genes are expressed in neurons^34^ and glia,^35,36^ particularly in structures that are targeted in neurodegenerative diseases.^34,35,37^ IGF and insulin are associated with regulating and maintaining cognitive function,^38^ and participate in neuronal and glial functions such as growth, metabolism, survival, gene expression, protein synthesis, cytoskeletal assembly, neurotransmitter function, synapse formation and plasticity.^34,39^

Glucose transporter 4 (GLUT4) is very important for glucose uptake and utilization in the brain.^38^ Insulin stimulates GLUT4 gene expression and protein trafficking from the cytosol to the plasma membrane, modulating glucose uptake and utilization.^38^ Consequently, the regulation of neuronal metabolism and the generation of energy needed for cognition and memory are linked to insulin stimulation of GLUT4.^38^ GLUT4 is abundantly expressed along with insulin receptors, in medial temporal lobe structures which are affected in AD pathology. Nevertheless, post-mortem brain studies have not detected significant reductions in GLUT4 expression in AD.^40^ Deficits in brain glucose utilization and energy metabolism, and brain insulin/IGF resistance could be mediated by impairments in GLUT4 trafficking between the cytosol and plasma membrane.^38^

Insulin and IGF binding to their own receptors activates some pathways, leading to phosphorylation and activation of intrinsic receptor tyrosine kinases. The phosphorylated receptors interact with IR substrate molecules and promote transmission of downstream signals that stimulate growth, survival, metabolism, plasticity and inhibit apoptosis.^38^

**Brain insulin/IGF resistance and AD.** AD has been associated with deficits in insulin/IGF signaling due to the effects of insulin/IGF resistance and deficiency.^5^ Deficits in cerebral glucose utilization have been described in the early stages of AD.^41-44^ Suzanne de la Monte and colleagues have proposed the concept of AD as "Type 3 diabetes".^40^ They observed an inverse correlation between IR abundance and the Braak score of AD brains, with 80% reduced IR substrates levels in the most severe cases. They described reduced messenger RNA levels of IGF-1 and increased Tau protein levels regulated by IR.^40,45^ Studies with small interfering RNA molecules showed that molecular disruption of brain insulin and IGF receptors was sufficient to cause cognitive impairment and hippocampal degeneration similar to AD molecular abnormalities.^46^

Brain insulin/IGF resistance/deficiency can appear independently of Type 1 and Type 2 diabetes.^5^ Neurodegeneration can occur by several mechanisms such as the activation of kinases that aberrantly phosphorylate tau, the expression of AβPP and accumulation of AβPP-Aβ in brain insulin/IGF resistance.^38^ Hyperglycemia leads to the accumulation of advanced glycation end products (AGEs) that disrupts removal of Aβ^42^ and induces Aβ and Tau glycation, promoting Aβ aggregation and NFTs formation in the brain.^38,47,48^ AGE production is found in normal aging, but becomes highly accelerated in diabetes.^49^ Recent evidence suggests that glyceraldehyde-derived AGEs (glycer-AGE) are the predominant modification of the most toxic forms of AGEs, and Glycer-AGE-modified proteins are directly toxic to cultured neurons. Diabetic serum enriched with glycer-AGE modified proteins has shown toxic effects on neurons.^10^ AGEs are also linked to microvascular alterations in hyperglycemia and diabetes.^50^ Receptor for advanced glycation end products (RAGE) expression has been associated with pathological conditions such as diabetic vascular disease, chronic inflammation and AD.^51,52^ Studies with immunohistochemistry for RAGE in AD brains have demonstrated that RAGE increased expression in neurons, microglia, astrocytes and vascular endothelial cells.^53,54^ RAGE binds and interacts with AGEs and also with Aβ.^49^ RAGE interaction with AGE-modified proteins in either diabetes or AD, or Aβ in AD, can produce damaging inflammatory responses^55,56^ and be responsible for vascular complications in DM and AD.^57-59^ RAGE mediates the transport of plasma Aβ across the BBB^60^ and the migration of monocytes across the human brain endothelial cells in response to Aβ.^61^

Microvascular disease is seen as a consequence of diabetes and can also be found in AD brains, possibly contributing to the cognitive impairment and neurodegeneration seen in AD.^5,62^ Decreased blood flow and impairment of oxygen and nutrient delivery exacerbate the adverse effects of insulin/IGF resistance.^63^ Consequently, there is an increase in oxidative stress and activation of signaling mechanisms which promote aberrant tau phosphorylation, AβPP cleavage, AβPP-Aβ deposition, and mitochondrial dysfunction.^38,63^

IR function is compromised in brain insulin/IGF resistance, leading to many adverse effects. There is decreased signaling through IR substrate, phosphoinositol-3-kinase (PI3K) and Akt, with reduced neuronal and oligodendroglial survival, neuronal plasticity and myelin maintenance.^38^ IR dysfunction increases activation of glycogen synthetase kinase 3β (GSK-3β) and phosphatases that negatively regulate insulin signaling, consequently producing increased tau phosphorylation, oxidative stress, neuro-inflammation and pro-apoptosis signaling.^38^ Reduced insulin-responsive gene expression seen in IR dysfunction can lead to deficits in acetylcholine and glucose metabolism.^38^

Impairment in GLUT4 functions in brain with insulin/IGF resistance results in reduced glucose uptake and utilization, consequently compromising cell energy and homeostatic functions, disrupting neuronal cytoskeleton and synaptic connection.^38^ Deficits in energy metabolism lead to increased oxidative and endoplasmic reticulum (ER) stress, and mitochondrial dysfunction with the generation of reactive oxygen (ROS) and reactive nitrogen species (RNS).^64-66^ Increased oxidative stress, ROS and RNS damage RNA, DNA, proteins, and lipid peroxidation production, energy deficits, cell death, increased AβPP expression, Aβ^42^ deposition and fibrillarization.^38^ There is activation of pro-inflammatory and pro-death cascades and down-regulation of target genes that mediate cholinergic homeostasis linked to AD in brain with insulin/IGF resistance.^5,67^ Impairment of myelin maintenance also occurs and can lead to increased neuro-inflammation, oxidative stress, pro-apoptosis, and further insulin resistance, besides white matter atrophy.^38^

The insulin-degrading enzyme (IDE) has the property of catabolizing insulin and Aβ, and may play a critical role in Aβ clearance in the brain as Aβ scavenger protease.^68,69^ IDE acts as a general regulator of amyloid burden in the pancreas and brain.^70^ Insulin regulates IDE expression and can directly compete with Aβ for binding to IDE.^71^ In hyper-insulin states, IDE can be diverted to degrade insulin, consequently allowing AβPP-Aβ accumulation.^70^ Mutations in the IDE gene in mice resulted in reduced activity of this enzyme, lower rates of Aβ and insulin degradation, additionally developing hyperinsulinaemia and accumulating Aβ species in their brains.^72^ Chronic hyperglycaemia, hyperinsulinaemia, oxidative stress, accumulation of AGEs, increased expression and activation of IDE, increased production of pro-inflammatory cytokines, and cerebral microvascular disease associated with peripheral insulin resistance could result in mild cognitive impairment and neurodegeneration.^38,73^

**Brain insulin/IGF resistance and Aβ pathology.** Altered proteolysis with increased AβPP gene expression results in the accumulation of 40 or 42 amino acid length Aβ peptides that can aggregate and have been described in AD pathology. Dysregulated expression and processing of AβPP leads to the accumulation of AβPP-Aβ oligomeric fibrils or insoluble larger aggregated fibrils in the form of plaques that are neurotoxic.^5^ The interest in the role of impaired insulin/IGF signaling as either the cause or consequence of dysregulated AβPP-Aβ expression and protein processing has grown in literature.^38^ Insulin can accelerate trafficking of AβPP-Aβ from the trans-Golgi network to the plasma membrane as well as its extracellular secretion^74^ and also inhibits its intracellular degradation by IDE.^75^ Impaired insulin signaling can disrupt both the processing of AβPP and clearance of AβPP-Aβ.^76^ Simultaneously, AβPP-Aβ affects insulin signaling by competing with insulin, or reducing the affinity of insulin for binding to its own receptor.^77^ AβPP-Aβ oligomers desensitize and reduce the surface expression of IRs, consequently inhibiting neuronal insulin-signaling.^67^ Additionally, intracellular AβPP-Aβ interferes with PI3k activation of Akt, leading to reduced signaling, increased activation of GSK-3β, and hyper-phosphorylation of tau. Increased levels of GSK-3 promote AβPP processing and AβPP-Aβ accumulation.^78^

**Brain insulin/IGF resistance and Tau pathology.** In AD, the main neuronal cytoskeletal lesions correlated with severity of dementia, including NFTs and dystrophic neurites, contain aggregated and ubiquitinated insoluble fibrillar tau.^4,38,79^ Tau gene expression and phosphorylation can be regulated by insulin/IGF stimulation.^80,81^ Reduced insulin/IGF signaling can impair tau gene expression and contribute to tau pathology.^82^ Brain insulin/IGF resistance results in decreased signaling through PI3K, Akt,^80,81^ and Wnt/β-catenin,^83^ and increased activation of GSK-3β.^84,85^ The hyper-phosphorylation of tau, which leads to tau misfolding and fibril aggregation in AD pathology, can be partly due to GSK-3β overactivation.^86^ Tau hyper-phosphorylation is mediated by increased activation of cyclin-dependent kinase 5 (cdk-5) and c-Abl kinases,^87,88^ and inhibition of protein phosphatases 1 and 2A.^88,89^ Tau protein misfolds and self-aggregates into insoluble fibrillar structures lead to neurofibrillary tangles, dystrophic neurites, and neuropil threads.^38,90^ The results of generation and accumulation of hyper-phosphorylated insoluble fibrillar tau are the exacerbation of cytoskeletal collapse, neurite retraction, and synaptic disconnection.^38^ Table 1 summarizes the main mechanisms linking brain insulin/IGF resistance to AD pathology.

**Table 1:** Summary of mechanisms linking brain insulin/IGF resistance to AD pathology.

Mechanisms	Consequences
Impairment of GLUT4 function	• Energy deficits: memory and cognition impairment; disruption of neuronal cytoskeleton and synaptic connection.
Changes in insulin receptor functions	• Increased activation of GSK-3and phosphatases: tau phosphorylation, oxidative stress, neuro-inflammation, pro-apoptosis signaling. • Decreased IR substrate, PI3K-Akt activity: reduced neuronal and oligodendroglial survival, neuronal plasticity, myelin maintenance. • Reduced insulin-responsive gene expression: deficits in acetylcholine and glucose metabolism. • Impairment in tau gene expression: hyper-phosphorylation of tau leading to tau misfolding and fibril aggregation, NFTs.
Energy deficit and hypometabolism	Increased oxidative and endoplasmic reticulum stress, and mitochondrial dysfunction with ROS and RNS generation.
Increased oxidative stress, ROS and RNS	Damaged RNA, DNA, proteins, and lipid peroxidation production, energy deficits, cell death, increased AbPP expression with Aβ42 deposition and fibrillarization.
hyperglycemia	Enhances AGE production and impairs RAGE expression: microvascular disease with brain hypoperfusion, inflammatory responses, impairment in removal of Aβ42 leading to Aβ42 deposition.

AD: Alzheimer disease; GLUT4 Glucose transporter 4; IR: insulin receptor; PI3K: phosphoinositol-3-kinase; NFTs: neurofibrillary tangles; ROS: reactive oxygen species; RNS: reactive nitrogen species; AβPP: amyloid beta precursor protein; Aβ42: amyloid beta 42; AGE: advanced glycation end products; RAGE: receptor for advanced glycation end products.

**Neurodegenerative process contributing to brain insulin resistance in AD.** Interestingly, the neuropathological process involved in AD can reinforce brain insulin resistance. Aβ toxicity, microvascular disease, oxidative stress, transition metal ion accumulations and hyperphosphorylated-ubiquitinated tau lead to increased brain insulin resistance.^38^ Aβ 42 toxicity competes with insulin and reduces the affinity of insulin binding to its receptor.^77,91^ AβPP oligomers desensitize and reduce surface expression of insulin receptors, and interfere with PI3K activation of Akt.^38,92^ The Aβ toxicity disrupts insulin signaling and impairs insulin stimulated neuronal survival and plasticity.^38^ Oxidative stress can produce increases in neuro-inflammation and pro-inflammatory cytokine inhibition of insulin signaling.^38^ Transition metal ion accumulations produce mitochondrial dysfunction, oxidative stress, tau and AβPP oligomer fibrillarization, which impair glucose uptake and utilization, and inhibit insulin signaling.^38^ Hyperphosphorylated-ubiquitinated tau increases oxidative stress, promotes neuroinflammation which consequently enhances insulin resistance.^38^ Microvascular disease exacerbates insulin resistance through cerebral hypoperfusion and hypoxic-ischemic injury.^38^

## Conclusions.

A body of evidence has shown that the structural and functional integrity of the CNS can be compromised in the presence of brain insulin and IGF resistance or deficiency. These changes can contribute to AD pathology and conversely, AD pathology can enhance brain insulin and IGF resistance, functioning as a positive feedback loop. However, it is necessary to bear in mind that the majority of studies have been conducted in the experimental field with animal or cell models. Elucidating the question of a connection among DM, brain insulin resistance/deficiency and AD is very important, especially for planning novel strategies to prevent and treat AD in the future.
